# Strategies for high-altitude adaptation revealed from high-quality draft genome of non-violacein producing *Janthinobacterium lividum* ERGS5:01

**DOI:** 10.1186/s40793-018-0313-3

**Published:** 2018-04-19

**Authors:** Rakshak Kumar, Vishal Acharya, Dharam Singh, Sanjay Kumar

**Affiliations:** Biotechnology Division, CSIR-Institute of Himalayan Bioresource Technology, Post BoxNo.06, Palampur, Himachal Pradesh 176 061 India

**Keywords:** *Janthinobacterium lividum*, Sikkim Himalaya, Comparative genomics, High-altitude adaptation

## Abstract

A light pink coloured bacterial strain ERGS5:01 isolated from glacial stream water of Sikkim Himalaya was affiliated to *Janthinobacterium lividum* based on 16S rRNA gene sequence identity and phylogenetic clustering. Whole genome sequencing was performed for the strain to confirm its taxonomy as it lacked the typical violet pigmentation of the genus and also to decipher its survival strategy at the aquatic ecosystem of high elevation. The PacBio RSII sequencing generated genome of 5,168,928 bp with 4575 protein-coding genes and 118 RNA genes. Whole genome-based multilocus sequence analysis clustering, in silico DDH similarity value of 95.1% and, the ANI value of 99.25% established the identity of the strain ERGS5:01 (MCC 2953) as a non-violacein producing *J. lividum*. The genome comparisons across genus *Janthinobacterium* revealed an open pan-genome with the scope of the addition of new orthologous cluster to complete the genomic inventory. The genomic insight provided the genetic basis of freezing and frequent freeze-thaw cycle tolerance and, for industrially important enzymes. Extended insight into the genome provided clues of crucial genes associated with adaptation in the harsh aquatic ecosystem of high altitude.

**Electronic supplementary material:**

The online version of this article (10.1186/s40793-018-0313-3) contains supplementary material, which is available to authorized users.

## Introduction

The genus *Janthinobacterium* was derived from the genus *Chromobacterium* (mesophilic, fermentative bacteria producing purple and violet colonies) to separate nonfermentative and psychrophilic bacteria producing violet colonies [1]. Hence, the most common feature of this genus is psychrophilic bacteria producing violet pigment violacein [1]. However, there have also been reports on partly pigmented and non-pigmented bacteria within this genus [1, 2]. In the present study, a light pink coloured bacterial strain ERGS5:01 isolated from glacial stream water sample in Sikkim Himalaya was affiliated to *Janthinobacterium lividum* by 16S rRNA gene sequence identity and phylogeny. The lack of typical violet pigmentation intrigued us to establish its taxonomic identity using whole genome sequencing. MLSA using multiple concatenated housekeeping genes was applied to investigate the phylogenetic position of the strain within *Janthinobacterium*. This method has been widely used to resolve the taxonomic position of closely related prokaryotic species within a genus [3]. The availability of whole genome sequences of multiple strains further allowed in silico DDH and ANI to confirm the taxonomic position of the strain with higher certainty [4].

The genus *Janthinobacterium* has a wide occurrence ranging from soil, aquatic sites, marine habitats, high altitude environments with a unique ability to survive and colonise new environments [5, 6].With the revolution in the field of microbial genomics and analyses such as pan-genome, it becomes handy to compare many strains of a species or genus to obtain a complete inventory of genes [7]. We used the genome sequence of strain ERGS5:01 and other strains to study the genomic diversity within this genus. The bacterial strain was isolated from an aquatic ecosystem of a high altitude region (4718 masl) [8]. Organisms in such environment sustain temperature fluctuation and are exposed to strong ultraviolet-B radiation, with low nutrient availability. [9, 10]. Bacterial cold associated adaptive traits to withstand such harsh conditions includes proteins required to maintain molecular central dogma and membrane fluidity at low temperature. [11]. Other associated proteins are those which response to osmotic, oxidative and cold stress [12]. The copy number of these proteins have often been reported to increase to accelerate the number of active sites to neutralise the lowered enzymatic rates at low temperatures by the cold-active organisms [13]. In the present study, we present an extended genomic insight of strain ERGS5:01 to explore their taxonomic position and to identify potentially important proteins for their survival in harsh environments of the high altitude aquatic ecosystem.

## Organism information

### Classification and features

**Fig. 1:**
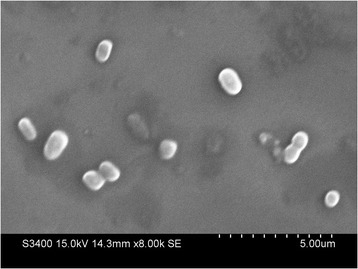
Scanning electron micrograph of strain *J. lividum* ERGS5:01 grown at ABM agar plates for 48 h at 10 ° C. for 15 days. Bar, 5 μm

The East Rathong glacier falls in the survey of India topo-sheet no. 78A/2 within the Khangchendzonga National Park area in the Sikkim Himalaya. It lies between 27°33′ and 27°36´ N latitude and 88°04 and 88°08′ E longitude in the West district of the state Sikkim in India [14]. During the isolation of psychrotrophs to explore for bioprospection, this aerobic chemoheterotrophic bacterial strain ERGS5:01 was isolated from a glacial stream located in the ablation zone of East Rathong glacier at an altitude of 4718 masl [8].The bacteria was isolated on ABM agar plates [peptone (0.5%, *w*/*v*), yeast extract (0.2%, w/v) and agar (2%, w/v)] [15] by incubating at 10 °C for 15 days. ERGS5:01 is a gram-negative, aerobic bacteria with optimum growth at 10 °C. The strain produced light pink colour colonies after a 72 h incubation at temperature 15 °C, 10 °C, and 4 °C. The colonies were found to be round, convex and entire. This bacteria could grow at the temperature range of 4–28 °C, NaCl concentration range of 1% to 4%, and pH range of 3–10 pH (Table 1). Scanning electron microscopy revealed the shape of the bacteria as short rods with an average length of 0.8 to 1.1 μm (Fig. 1).

**Table 1:** Classification and general features of *Janthinobacterium lividum* ERGS5:01 [18]

MIGS ID	Property	Term	Evidence code^a^
	Classification	Domain *Bacteria*	TAS [65]
		Phylum *Proteobacteria*	TAS [66]
		Class *Betaproteobacteria*	TAS [67]
		Order *Burkholderiales*	TAS [68]
		Family *Oxalobacteraceae*	TAS [69]
		Genus *Janthinobacterium*	TAS [69]
		Species *lividum*	TAS [69]
		strain: ERGS5:01(Accession MCC 2953)	IDA
	Gram stain	Negative	IDA
	Cell shape	Short rods	IDA
	Motility	Motile	IDA
	Sporulation	Nonsporulating	IDA
	Temperature range	4–28 °C	IDA
	Optimum temperature	10 °C	IDA
	pH range; Optimum	3–10;7	IDA
	Carbon source	Xylose, Maltose, Fructose, Dextrose, Raffinose, Trehalose, o-nitrophenyl-β-D-galactoside, Esculin	IDA
MIGS-6	Habitat	Water, Glacial stream	IDA
MIGS-6.3	Salinity	1% to 4%NaCl	IDA
MIGS-22	Oxygen requirement	Aerobic	IDA
MIGS-15	Biotic relationship	free-living	IDA
MIGS-14	Pathogenicity	NonPathogenic	NAS
MIGS-4	Geographic location	West Sikkim, India	IDA
MIGS-5	Sample collection	02-May-2015	IDA
MIGS-4.1	Latitude	27°33′15″ N	TAS [6],IDA
MIGS-4.2	Longitude	88°07′406″E	TAS [6],IDA
MIGS-4.4	Altitude	4718	TAS [6], IDA

^a^Evidence codes - *IDA* Inferred from Direct Assay, *TAS* Traceable Author Statement (i.e., a direct report exists in the literature), *NAS* Non-traceable Author Statement (i.e., not directly observed for the living, isolated sample, but based on a generally accepted property for the species, or anecdotal evidence). These evidence codes are from the Gene Ontology project [70]

#### Extended feature descriptions

##### 16S rRNA gene analysis

**Fig. 2:**
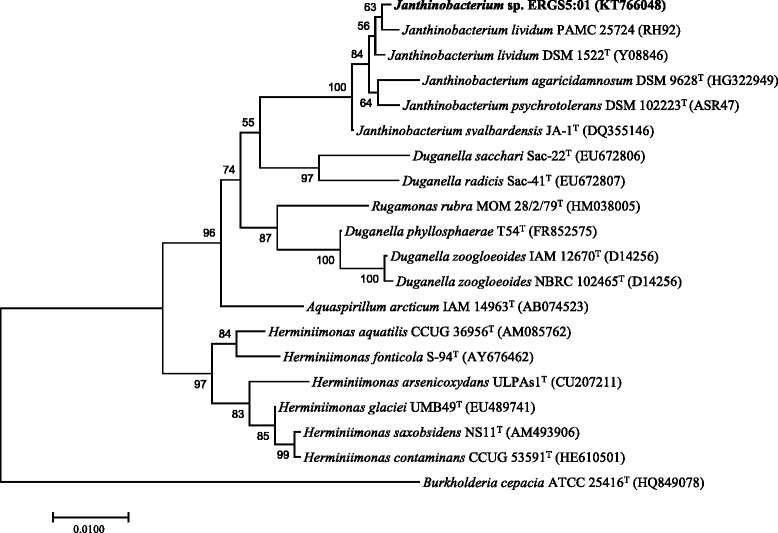
Phylogenetic tree based on 16S rRNA gene sequences of strain *J. lividum* ERGS5:01and nearest validly published strains obtained from NCBI database [16] was created using MEGA version 7.0 [17]. The sequences were aligned using Clustal W and the Neighbor-Joining tree was created based on the Jukes-Cantor model with 1000 bootstrap replications. Strain ERGS5:01 forms cluster with *J. lividum* PAMC 25724^T^*. Burkholderia cepacia* ATCC 25416^T^ was used as the out group organism and the scale bar corresponds to the expected number of changes per nucleotide position. NCBI accession numbers are given in parenthesis

Sequence identity search based on 16S rRNA gene sequence (1341 bp/ NCBI Accession No. KT766048) of strain ERGS5:01 with a database of type strains as available in NCBI [16] exhibits closest sequence identity of 99% with *J.* *lividum* PAMC 25724. Phylogenetic clustering constructed using Neighbor-Joining tree using Jukes-Cantor model of sequence evolution with 1000 bootstrap replications using Molecular Evolutionary Genetics Analysis version 7.0 [17] also clustered the strain ERGS5:01 with *J.* *lividum* PAMC 25724 (Fig. 2).

##### Biochemical profiling, extracellular enzyme assay, freezing and freeze-thaw tolerance

**Fig. 3:**
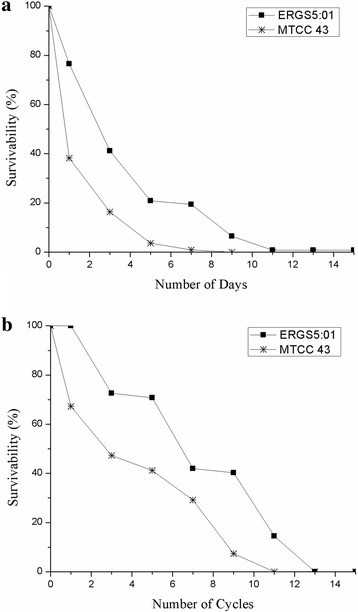
Survival of strain *J. lividum* ERGS5:01 to freezing (**a**) and freeze-thaw cycle (**b**)using colony count method considering the count of unfrozen sample as 100%. **a** Freeze tolerance up to 15 days at − 20 °C. **b** Freeze-thaw cycle tolerance up to 15 cycles with cycle consisting of 1 h of freezing at − 20 °C and thawing for 1 h at 10 °C . Mean of triplicate colony count were used to calculate survival percentage and strain *E. coli* MTCC43 was used as negative control

The strain ERGS5:01 was tested for various biochemical activities such as catalase, oxidase, triple sugar iron, citrate utilisation, urease, indole, MR-VP, motility and carbohydrate utilisation (KB009 HiCarbohydrate™ kit, HiMedia).The strain was observed as gram-negative short rods, motile, non-fermentative, positive in oxidase, catalase and urease and negative in MR-VP test. Out of the 35 sugars tested, this strain could utilize xylose, maltose, fructose, dextrose, raffinose, trehalose, o-nitrophenyl-β-D-galactoside, esculin while it could not utilize lactose, galactose, melibiose, sucrose, L-arabinose, mannose, inulin, sodium gluconate, glycerol, dulcitol, inositol, sorbitol, mannitol, adonitol, arabitol, erythritol, α-methyl-D- glucoside, ribose, rhamnose, cellobiose, melezitose, α-methyl-D-mannoside, xylitol, D-arabinose, malonate and, sorbose. The extracellular enzymatic activities namely amylase, lipase, protease and cellulase for strain ERGS5:01 were analysed using standard plate assay at 10 °C. The strain showed positive results for amylase, lipase, and protease activities. Survival percentage for freezing and frequent freeze-thaw cycle tolerance was tested by colony count method considering count on day 0 as 100% as described by Shivaji et al. [15]. For freeze tolerance, 27 tubes of 1 ml culture were allowed to reach stationary phase using ABM broth, and 24 of them were placed at − 20 °C. At each time point (1,3,5,7,9,11,13,15 days of freezing), three tubes were removed, thawed for 1 h at 10 °C and 100 μl were serially diluted in 900 μl of 0.9% saline. Three unfrozen tubes served as zero time point. The diluted culture was plated on ABM agar and incubated for 3–5 days at 10 °C. The mean from triplicate colony counts results were used for determining the survival percentage considering the cell count on day 0 as 100%. For freeze-thaw cycle tolerance, a similar procedure as described for freezing tolerance was followed, but freezing and thawing were in continuous cycles (1,3,5,7,9,11,13,15 cycles). Each cycle comprised of freezing at − 20 °C for 1 h followed by thawing at 10 °C for 1 h. We used *E. coli* MTCC 43 as a negative control because the strain showed optimum growth at 37 °C. The strain survived the freezing temperature of − 20 °C for 30 h as no ice crystal formation was observed in the culture broth. The percentage survivability of our strain was observed to be 76.60% for day 1. Subsequently, the survivability decreased to 41.12%, 20.9% and 19.35% on day 3,5 and 7 respectively. Further, from the 9th day, we observed growth below 10% growth. On the other hand strain, *E. coli* MTCC43 showed a steep decline of 40% within 24 h of incubation and declined steadily in successive cycles with zero survivability from day 9 (Fig. 3a). The strain showed higher resistance to the freeze-thaw cycle compared to freezing. The strain survived 100% at the first cycle which further reduced to 72.58%, 70.77%, 41.93%, 40.3% and 14.5% at subsequent cycle 3,5,7,9 and 11, respectively. Growth was observed to be seized on the 13 and 15 cycles. However, the survival of strain *E. coli* MTCC43 declined steadily to 67.27% in cycle 1 which further declined to 47.27%, 41.12%, 29.09% and 7.3% at subsequent cycles 3,5,7 and 9 respectively. Growth was observed to be seized at the cycles 11, 13 and 15 (Fig. 3b).

## Genome sequencing information

### Genome project history

The whole genome of the strain ERGS5:01 was sequenced owing to its lack of usual violet pigmentation, typical to the genus *Janthinobacterium*, and their ability to survive harsh aquatic ecosystem of the high altitude region. The work was carried out as a part of a project to understand the genetic basis of survival of psychrotrophs and its bioprospection from East Rathong Glacier in the Sikkim Himalaya. The sequencing was completed at CSIR-Institute of Himalayan Bioresource Technology, Palampur using PacBio RS II platform (Microsynth AG, Switzerland). The draft genome has been deposited in GenBank under the accession MAQB00000000 while the version described in this paper is MAQB02000000. The project summary with minimum information about a genome sequence [18] is shown in Table 2.

**Table 2:** Project information

MIGS ID	Property	Term
MIGS 31	Finishing quality	Level 2: High Quality Draft
MIGS-28	Libraries used	SMRTbell Template Prep kit v1.0 (20 kb)
MIGS 29	Sequencing platforms	PacBio
MIGS 31.2	Fold coverage	38.09×
MIGS 30	Assemblers	Hierarchical Genome Assembly Process v. 3
MIGS 32	Gene calling method	Prodigal
	Locus Tag	BA896
	Genbank ID	MAQB00000000
	GenBank Date of Release	October 16, 2016
	GOLD ID	Gp0177310
	BIOPROJECT	PRJNA327173
MIGS 13	Source Material Identifier	MCC 2953
	Project relevance	High altitude environment

### Growth conditions and genomic DNA preparation

The strain ERGS5:01 was regularly grown at 10 °C in ABM agar. Genomic DNA from the strains was extracted using GenElute™ Bacterial Genomic DNA Kit (Sigma-Aldrich, US).The obtained genomic DNA was evaluated for its quality and quantity using 1% agarose gel electrophoresis and Qubit 2.0 Fluorometer (Invitrogen, USA).

### Genome sequencing and assembly

Shearing of genomic DNA (10 μg) was done using g-TUBE™ (Covaris, US) and DNA library was prepared using 10 kb insert size with PacBio SMRTbell library preparation kit v1.0 [6]. Quantification of the prepared library was done using Qubit 2.0 Fluorometer (Invitrogen, USA). Sequencing was performed using PacBio RSII system (Pacific Biosciences, US) as described previously [19, 20]. Assembly of the generated subreads was performed de novo using RS hierarchical genome assembly process protocol version 3.0 (HGAP.3) in SMRT Analysis version 2.3.0 (Pacific Biosciences, US).

### Genome annotation

Annotation of the high-quality draft genome was performed using the JGI Prokaryotic Automatic Annotation Pipeline [21] with the additional analysis and the manual review being done within the IMG platform [22, 23]. The functions of the predicted protein-coding genes and genes with Pfam domains were assigned using the Interpro platform [24]. Genes assigned to COGs were assigned by searching against COG database (from the NCBI conserved domain database [25]) using rpsblast with significant E-value of 0.0001.BLASTclust with thresholds of 70% covered length and 30% sequence identity was used to obtain the number of genes in internal clusters [26]. Signal peptides and transmembrane helices were predicted using SignalP [27] and TMHMM [28] respectively. CRISPR database was used to identify CRISPR repeats in the genome [29].

## Genome properties

**Fig. 4:**
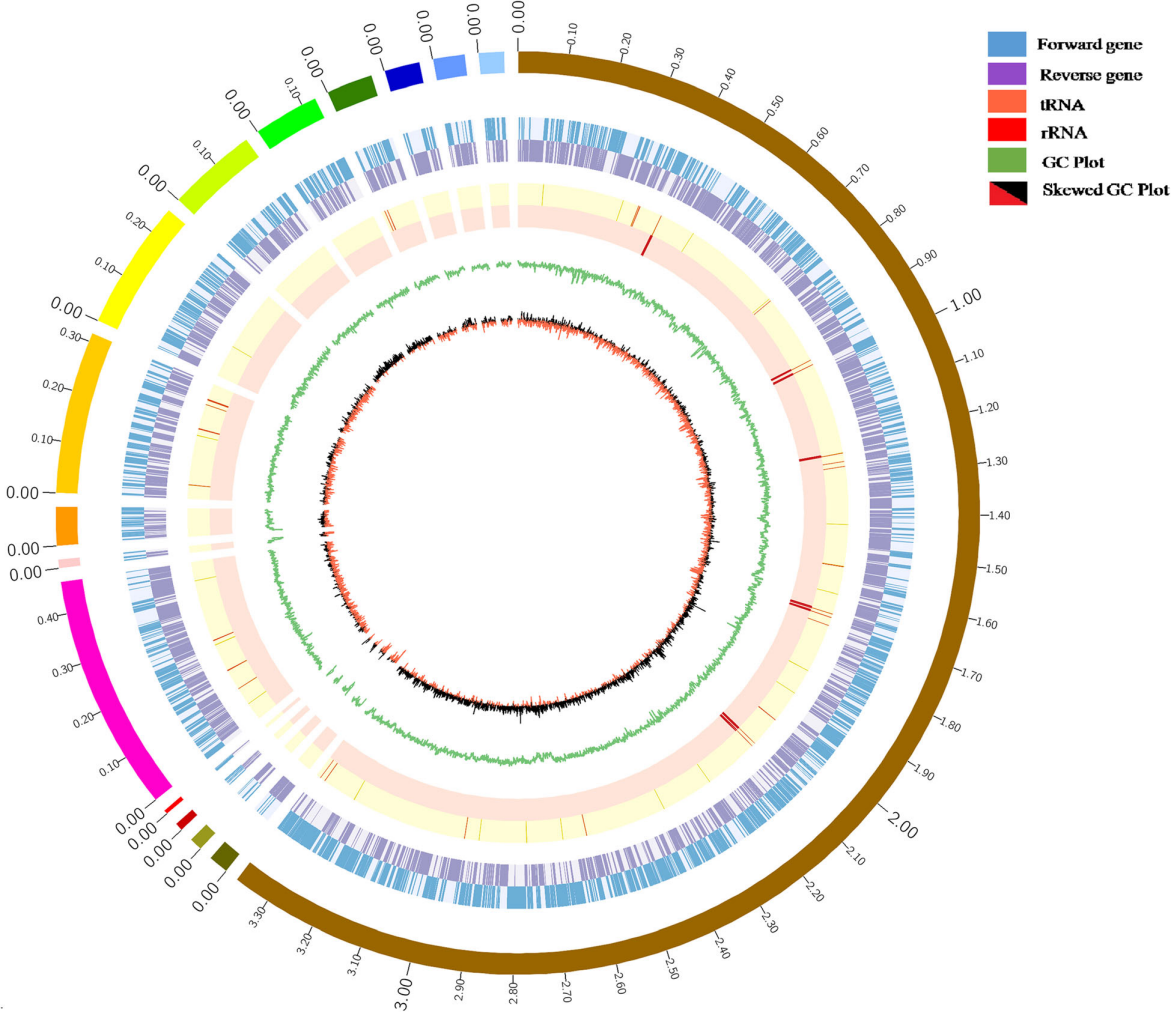
A circular chromosomal map of the draft genome of strain *J. lividum* ERGS5:01

The strain ERGS5:01 was assembled into 16 contigs containing the genome of total 5,168,928 bp with a G + C content of 60.48% (N50 contig length of 3,372,370 bp with average reference coverage of 38.09 X). A total of 4693 genes were predicted out of which 4575 were protein-coding genes, 118 were RNA genes (25 rRNAs, 90 tRNAs, and three non-coding RNAs) and 600 pseudo genes (Table 3). The circular chromosomal map for the draft genome is presented in Fig. 4 using ClicO FS, an online service based on Circos [30]. From COG database, 2559 genes were assigned to biological functions and 3160 genes (67.33%) were reported to be assigned to protein families. Table 3 summarises the genome properties and statistics, and Table 4 presents the distribution of genes into COG functional categories.

**Table 3:** Genome statistics

Attribute	Value	% of Total
Genome size (bp)	5,168,928	100.00%
DNA coding (bp)	4,513,533	87.32%
DNA G + C (bp)	3,126,125	60.48%
DNA scaffolds	16	100.00%
Total genes	4693	100.00%
Protein coding genes	4575	97.49%
RNA genes	118	2.51%
Pseudo genes^a^	600	12.78%
Genes in internal clusters	805	17.15%
Genes with function prediction	2988	63.67%
Genes assigned to COGs	2559	54.53%
Genes with Pfam domains	3160	67.33%
Genes with signal peptides	435	9.27%
Genes with transmembrane helices	908	19.35%
CRISPR repeats	0	0.00%

^a^Pseudogenes may also be counted as protein coding or RNA genes, so is not additive under total gene count

**Table 4:** Number of genes associated with general COG functional categories

Code	Value	%age	Description
J	187	6.51%	Translation, ribosomal structure and biogenesis
A	1	0.03%	RNA processing and modification
K	207	7.21%	Transcription
L	91	3.17%	Replication, recombination and repair
B	2	0.07%	Chromatin structure and dynamics
D	27	0.94%	Cell cycle control, Cell division, chromosome partitioning
V	66	2.30%	Defense mechanisms
T	240	8.35%	Signal transduction mechanisms
M	198	6.89%	Cell wall/membrane biogenesis
N	140	4.87%	Cell motility
U	76	2.65%	Intracellular trafficking and secretion
O	131	4.56%	Posttranslational modification, protein turnover, chaperones
C	144	5.01%	Energy production and conversion
G	130	4.52%	Carbohydrate transport and metabolism
E	221	7.69%	Amino acid transport and metabolism
F	71	2.47%	Nucleotide transport and metabolism
H	145	5.05%	Coenzyme transport and metabolism
I	121	4.21%	Lipid transport and metabolism
P	147	5.12%	Inorganic ion transport and metabolism
Q	51	1.78%	Secondary metabolites biosynthesis, transport and catabolism
R	216	7.52%	General function prediction only
S	153	5.33%	Function unknown
–	2134	45.47%	Not in COGs

The total is based on the total number of protein coding genes in the genome

## Insights from the genome sequence and comparative genomics

**Fig. 5:**
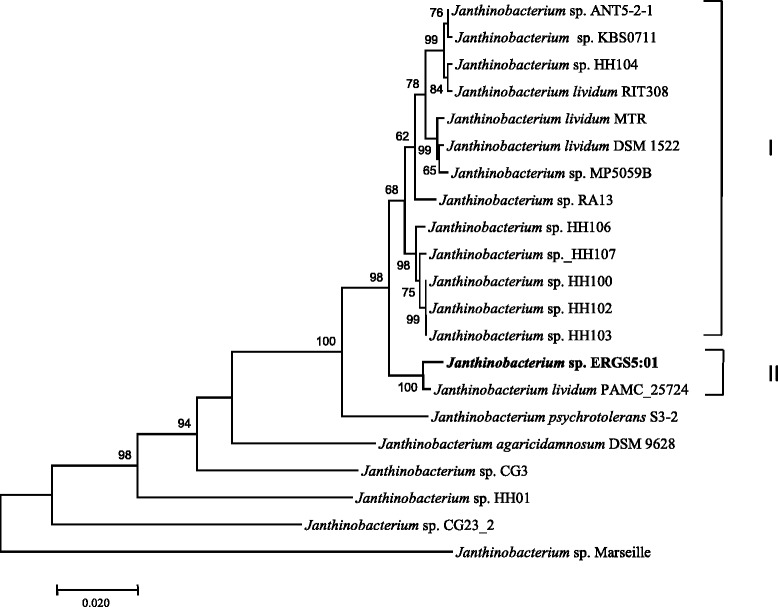
Multilocus sequence analysis (MLSA) clustering based phylogenetic tree of six concatenated housekeeping genes as derived from the whole genome sequence from the strains of *Janthinobacterium.* The tree was constructed using the maximum likelihood method based on the JTT matrix-based model using MEGA7.Bootstrap values over 50% (1000 replications) were shown at each node. All positions containing gaps and missing data were eliminated. Among *J*. *lividum*, two clusters were formed; cluster I showed the presence of vioacelin-containing genes whereas cluster II lacked it

The strain ERGS5:01 appeared sister to *J.lividum* PAMC 25724 based on its 16S rRNA gene sequence identity and phylogeny (Fig. 2). However, the 16S rRNA gene sequence identity between other species of *Janthinobacterium* also showed an identity above the threshold value (> 98.7%) (Table 5) as recommended for species identity by Meier-Kolthoff et al. [31]. The insufficiency of 16S rRNA genes in resolving species for many genera [3] led us further explore the phylogenetic position of the strain ERGS5:01 using six housekeeping genes namely, *rpoB*, *aroE*, *gmk*, *RecA*, *gyrB* and *tpi*. These genes were retrieved from whole genome sequence available from strain ERGS5:01 and other 20 *Janthinobacterim* strains. Multiple alignments were performed using MAFFT, statistics for each locus was summarised using MEGA 7, and phylogenetic tree of concatenated six housekeeping genes was constructed using maximum likelihood method based on the JTT matrix-based model in MEGA 7 [32]. Neighbour-joining tree constructed with six concatenated housekeeping genes for MLSA analysis agreed with the data generated by the maximum likelihood method described above (Additional file 1: Figure S1).The MLSA clustering revealed monophyly of strain ERGS5:01 and *J. lividum* PAMC 25724 (Cluster II) coherent to the 16S rRNA phylogeny (Fig. 5). This group formed a sister clade with other *J. lividum* strains with strong bootstrap support of 98% (Cluster I) (Fig. 5). Such distinct separation among *J. lividum* strains prompted us to carry out an exhaustive automatic BLAST as well as manual searches to elucidate the presence of *vioABCDE* operon genes among genomes available from *J. lividum* strains. Interestingly, the distinct violacein pigment (from which the genus *Janthinobacterium* derives its name) producing genes were absent in both the strains of cluster II and all the strains among cluster I contained *vioABCDE* operons (Fig. 5). Hence, the separation of two clusters among J. lividum strains was based on possession of *vioABCDE* operons. We then performed whole genome sequence-based in silico DDH using the online genome-to-genome calculator with the GGDC 2.0 BLAST+ model [33] and ANI using nucleotide fasta sequences of each genome compared to the genome of strain ERGS5:01 as a reference with the Perl script [34]. The observed DDH value was 95.15% and, ANI value was 99.25% between strain ERGS5:01 and PAMC 25724 (Table 5 and Additional file 2: Table S1). Both the values qualify above the cut-off value for species boundary [33, 34], and hence the results were consistent with the MLSA clustering of strain ERGS5:01 with PAMC 25724. In a recent study, strain PAMC 25724 has been reported as the strain of the species *J. lividum* [35] and is a validly published species with the availability of culture at Polar and Alpine Microbial Collection with accession number 25724. In another study, strains HH100, HH102, HH103, HH104, HH106, HH107, and 5059B had shown to be clustered together in proximity to *J. lividum* DSM 1522 [6]. Based on DDH and ANI values and MLST clustering, the strain ERGS5:01 could be affiliated to a non-violaecin producing strain of *J. lividum*. We also propose that strain ERGS5:01 and PAMC 25724 has the scope of reassessment of their taxonomic position under *J. lividum* that will require further comparative polyphasic taxonomic studies. The strain ERGS5:01 is deposited in the MCC at National Centre for Cell Science, Pune, India with accession number MCC 2953 (Additional file 3: Figure S2).

**Table 5:** Sequence identity of *Janthinobacterium lividum* ERGS5:01 with validly described species of genus *Janthinobacterium*; 16S rRNA gene sequence identity and whole genome sequence-based in silico comparison for DDH and ANI

Strain Name	16S rRNA gene identity^a^	DDH (Model-based Confidence Interval) (%)	ANI (%)
*Janthinobacterium lividum* PAMC25724	99%	95.10 (93.5–96.3)	99.25
*Janthinobacterium lividum* DSM 1522	99%	38.30 (35.9–40.9)	89.53
*Janthinobacterium psychotolerans* S3–2	99%	26.80 (24.4–29.3	82.13
*Janthinobacterium agaricidamnosum* DSM 9628	99%	23.00 (20.7–25.5)	78.86

^a^Values for 16S rRNA identity are based on NCBI BLAST alignment of the ERGS5:01 16S rRNA gene against genomes in IMG

The psychrotrophic strain ERGS5:01 isolated from the glacial stream was found to tolerate freezing as well as frequent freeze-thaw cycles. The phenomenon is corroborated by the presence of numerous genes encoding for proteins responsible for cold adaptation. Genes encoding for cold-shock proteins; multi-enzyme complex (UvrD helicase, UvrABC helicase, UvrB/UvrC); DNA repair proteins (RecN, RecO, RadA, MutS, deoxyribose dipyrimidine photolyase); and cluster of chaperone proteins (ClpB, DnaK, DnaJ, Hsp31, HtpG, SurA, HscA, EcpD and FliS) were observed in the genome of strain ERGS5:01. An elaborate discussion on such genes associated with cold adaptation from the strain is presented under extended insight section of this paper. On plate assay, the strain was observed to produce cold active extracellular enzymes namely amylase, lipase, and protease. Hence, the genomic data were checked for the presence of potential industrially important enzymes using data mining. Genomic data supported the plate assay results as we obtained one copy of alpha-amylase and serine protease along with three copies of lipase-encoding genes (Additional file 4: Table S2). The genomic data revealed multiple copies of some other noted industrially important enzymes like phospholipase, glycosyl transferase, alcohol dehydrogenase, catalase, alkaline phosphatase and chitinase (Additional file 4: Table S2).

Comparative genomic study was performed to reveal the genomic diversity across the genus Janthinobacterium*.* Genome data included *J. agaricidamnosum* DSM9628 (HG322949, [36]); *J. psychotolerans* S3–2 (LOCQ00000000, [35]) and *J. lividum* with multiple strains of Marseille (CP000269; [37]); Ant5–2 (LNCE00000000, [38]); PAMC 25724 (AHHB00000000, [2]; CG3 (APFF00000000, [39]); HH01 (NZ_AMWD00000000, [40]); RIT308 (JFYR00000000, [41]); MTR (JRRH00000000, [42]);RA13 (JQNP01000001, [43]); KBS0711 (LBCO00000000, [44]); CG23_2 (CYSS00000000, [45]); DSM1522 (LRHW00000000), MP5059B (LRHX00000000), HH100 (LRHY00000000), HH102 (LRHZ00000000), HH103 (LRIA00000000), HH104 (LRIB00000000), HH106 (LRIC00000000), and HH107 (LRID00000000) [4]. Few other genome sequence were retrieved from Gold database or IMG [22, 23]: *J. lividum* strain NFR18 (FPKH00000000); *Janthinobacterium* sp. strains 551a (FMXD01000000), OK676 (FNHA01000000), 344 (FOKL01000001.1), TND4EL3 (FTMV00000000) and YR213(FNDA01000000).

The genome-wide amino-acid analysis of all 27 psychrotolerant *Janthinobacterium* strains revealed broad similarities in the usage profiles of Ala, Leu, Gly, and Val as the most frequently used amino acids. An ultra-fast computational pipeline Bacterial Pan Genome Analysis Tool [46] was used to assess all 27 genomes for comprehensive pan-genome studies based on power law model. The pan-genome curve perfectly fits a power law function with an exponent of 0.447968 indicating that the pan-genome of the genus *Janthinobacterium* is open (Additional file 5: Figure S3). Greater than zero exponents and, open pan-genome correspond to the incomplete gene inventory with the scope of the additions of new orthologous clusters [7, 47]. The orthologous gene cluster for the pan-genome (complete gene family) was observed to be 21,349 out of which 1066 (~ 5%) were core genome. Core genomes represent the list of gene families shared by all 27 *Janthinobacterium* genomes. All the strains reported under the genus were psychrotolerant. Interestingly, we obtained various categories of genes associated with cold adaptation within the list of core genomes (namely, two-component histidine kinase, cold-shock proteins, cold-active chaperone, DNA repair, carbon storage/starvation, membrane/cell wall alteration and oxidative stress) (Additional file 6: Table S3). Among the core genomes of 27 strains, ~ 95% of the genes could be assigned to COG categories (Additional file 7: Table S4). The highest percentage of the genes (17.5%) in these COG categories were associated with signal transduction mechanism (Additional file 7: Table S4). Likewise, a recent report on *Pseudoalteromonas haloplanktis* TAC125 has discussed the role of major stimulus signalling transduction cascades–TCS histidine kinase on the bacterial adaptation to cold and deep water [48]. Total numbers of accessory genes observed were 20,283 which include the species-specific unique genes ranging from 0 to 2860 genes (Additional file 5: Figure S3). The open pan-genome with the discrepancy in the number of unique genes among strains strongly supports the high diversity in the genomic cluster of the genus *Janthinobacterium*. Importantly, strain CG23_2 which showed a maximum number of unique genes also has the largest genome in the genus *Janthinobacterium* [45]. All reported strains are psychrotolerant and have diverse habitat range as supported by the diversity in genomic structure revealed by the pan-genome analysis.

### Extended genomic insights into adaptation to the high altitude aquatic environment

Exhaustive data mining across the genome of strain ERGS5:01 was carried out to identify potential genes responsible for its assistance in the survival of aquatic high altitude environment. Multiple copies of genes for cold adaptation and other stress response proteins were observed as discussed below.

#### Two-component systems (TCS) histidine kinase and, signal transduction pathways

TCS are widespread in bacteria, and used for monitoring and adapting to changes in their extra- or the intracellular environment. Various chemical and physical stimuli including pH, temperature, oxidative stress induce differential expression of TCSs in bacteria [49]. Furthermore, this two-component histidine-kinase system has been reported for their role in the bacterial survival at cold [50, 51]. The genome of strain ERGS5:01 contained 58 copy numbers of such TCS. The report on blockage of cold-sensitive secretion pathway in *E. coli* has revealed the critical role of signal peptide/ secretion route for growth at low temperature with the aquatic environment [50]*.* Flagellin-specific chaperone (FliS) which binds to flagellin and facilitates bacterial transport was also observed in strain ERGS5:01. This observation further supports the presence of signal transduction and secretory pathways essential for survival at cold-temperature aquatic conditions.

#### Pigmentation

Pigmentation of bacteria is reported to play an important role in cold and radiation adaptations [7].The strain ERGS5:01 lacked the usual violet pigment of the genus, and likewise there was no observation of violacein -producing gene (*vioA*, *vioB*, *vioC*, *vioD*, and *vioE*) in the genome. However, it produced the light pink pigment that intrigued us to explore the genome for genes involved in carotenoid/ terpenoids biosynthesis pathway. Two copies of phytoene synthase genes and, one copy each of phytoene desaturase, phytoene dehydrogenase, lycopene beta-cyclase, octaprenyl diphosphate synthase, and dimethyl alanine transferase were observed. The presence of carotenoid/terpenoids biosynthesis pathway genes may assist this strain in providing tolerance to UV-B radiations, maintaining homeostasis during temperature fluctuations and adaptability in harsh condition of glacial ecosystems [9]. Multiple copies of genes like UvrD helicase (5), UvrABC helicase (1) and, UvrB/UvrC (1) were also observed which may assist against UV damage.

#### Oxidative stress response

High exposure to UV radiations causes damage to bacteria surviving in extremely high altitude conditions by generating free radicals [52]. Increase in oxidative stress in *Pseudomonas fluorescens* MTCC 667 grown at low temperature was reported by the enhanced level of enzyme (namely, superoxide dismutase and catalase) activities and free radicals. [53]. Elevated activity of another antioxidant enzyme thioredoxin reductase in *Listeria monocytogenes* growing at 10 °C as compared to a reference culture grown at 37 °C was also reported [54]. We observed numerous copies of putative oxidases genes in strain ERGS5:01 that leads to the production of large quantities of intrinsic H_2_O_2_ and other reactive oxygen species. The genome includes multiple copies of the thioredoxin (10), peroxiredoxin (5), alkyl hydroperoxide reductase (3), organic hydroperoxide reductase (2) and, a copy each of thioredoxin reductase, superoxide dismutase, catalase-peroxidase genes.

#### DNA repair and cold-shock chaperones

It has been well-reviewed and demonstrated that CSPs are strongly induced in bacteria in response to a rapid decrease in growth temperature [55–57]. CSPs are involved in RNA metabolism which prevents secondary structure formation and facilitates degradation of structured RNA, hence functioning as RNA chaperones. Two copies of genes inducing CSPs have been observed in the strain ERGS5:01 which potentially assist in the tuning of RNA metabolism in the cold adaptation. Increased expression of the *HtpG* and *GroEL* gene have been observed in response to low temperatures in cyanobacterial strains, *Synechococcus* sp. PCC 7942 [58]. Similarly, strain ERGS5:01 also possess a single copy of *HtpG*, *GroEL*, and *GroES* genes which may involve in the acclimation to low temperatures.

#### Horizontal gene transfer (HGT) supporting adaptation to cold

Numerous bacteria, unlike eukaryotes, have acquired a significant portion of DNA from distantly related organisms [59]. Such acquisitions have been reported to be prevalent in the prokaryotic genome with a low frequency of recombination and have greatly increased the genomic diversity, enabling bacteria to adapt and colonise in extreme and hostile conditions [60]. This phenomenon prompted us to investigate the occurrence of any such horizontally acquired genes in strain ERGS5:01 that may confer help in its adaptation to extreme conditions. Entire genes of strain ERGS5:01 were queried against the locally constructed database of other 19 *Janthinobacterium* genomes using BLAST with significant E-value of 1e-15. These blast results provided the list of 12 genes with no match against any available *Janthinobacterium* genomes indicating possibilities of HGT (Table 6). The G + C compositions of these genes were also indicative of HGT as it varied from the usual 60% G + C of genus *Janthinobacterium* (Table 6) [60, 61]. The HGT-acquired genes include two copies of glycosyl transferase family that participates in peptidoglycan biosynthesis involved in providing the protective shell around bacterial cell membranes and in cell elongation and cell division [62]. This enzyme has been reported to have increased expression in *Shewanella oneidensis* at low temperature [63] and might be considered as one of the crucial genes in the strain ERGS5:01for cold adaptation. Another important gene encoding for tellurium resistance protein namely Ter C, a general stress response protein was also observed. In spite of the fact that some other *Janthinobacterium* genomes also possess genes encoding for TerC, yet the gene observed in strain ERGS5:01 suggested different amino-acid composition that was more closely related to *Variovorax paradoxus* with a sequence similarity of 84%. Further studies are necessary to ascertain their specific role(s) for cold adaptation in strain ERGS5:01.

**Table 6:** Putative horizontally acquired genes of strain ERGS5:01; their closest match, gene length and G + C% composition

Sl No.	Name of gene (GenBank No.)	Closest match in NCBI GenBank/percentage similarity (Phylum)	DNA (bp)	G + C (%)
1	Transcriptional regulator (OFJ47798)	*Lysobacter/*74% (*Gammaproteobacteria*)	252	50.39
2	Glycosyltransferase family 1 (OFJ49554)	*Polaromonas napthalenivorans/*79% (*Betaproteobacteria*)	1155	56.88
3	Integrase (OFJ49513)	*Burkholderia ubonensis*/73% (*Betaproteobacteria*)	1050	50.19
4	Single-stranded DNA-binding protein (OFJ48621)	*Burkholderia thailandensis/* 84% (*Betaproteobacteria*)	339	53.39
5	Terminase (OFJ46446)	*Brachymonas chironomi*/75% (*Betaproteobacteria*)	519	58.38
6	Sulphate transporter (OFJ46406)	*Pseudomonas fluorescens*/81% (*Gammaproteobacteria*)	624	59.29
7	Phage tail tape measure protein (OFJ46394)	*Acinetobacter* sp. NIPH 2168 /56% (*Gammaproteobacteria*)	1953	58.26
8	DNA-binding protein (OFJ50138)	*Acidovorax avenae/* 71% (*Betaproteobacteria*)	1131	47.3
9	Tellurium resistance protein TerC (OFJ50276)	*Variovorax paradoxus*/ 84% (*Betaproteobacteria*)	2064	52.4
10	Glycosyltransferase family 2 (OFJ50221)	*Pseudoduganella violaceinigra*/81% (*Betaproteobacteria*)	933	49.9
11	Flagellar motor protein MotB (OFJ49983)	*Variovorax paradoxus/*86% (*Betaproteobacteria*)	717	49.79
12	Acetyltransferase (OFJ50217)	*Xanthomonas axonopodis*/ 72% (*Gammaproteobacteria*)	678	46.01

## Conclusion

Sikkim Himalaya possesses untapped microbial resources with the tremendous scope of bioprospection [64]. Strain ERGS5:01 is one such light pink pigmented bacteria identified as *J. lividum*. The taxonomic identity of the strain remained uncertain as it lacked the usual violet pigmentation typical of the genus *Janthinobacterium*. Whole genome sequencing of the strain was performed owing to the discordance between unusual pigmentation and taxonomy and, survival at the harsh aquatic ecosystem. A high-quality draft genome of 5.1 Mb was generated and deposited at GenBank under accession No. MAQB00000000. MLSA clustering allowed better phylogenetic resolution while genome based GGDH and ANI supported the clustering and confirmed the identity of strain as a non-violecin producing *J. lividum*. Further, strain ERGS5:01 was studied for its biochemical and physiological features for adaptational strategies such as freeze and freeze-thaw tolerance. The comparative pan-genome analysis revealed an open-pan genome with the scope of the addition of new orthologous cluster to complete the inventory of genes of *Janthinobacterium* and, the discrepancy in the number of unique genes among strains strongly supported the high diversity in the genomic cluster of this genus*.* The genomic insight of strain ERGS5:01 provided a genetic basis for its tolerance to freezing and frequent freeze-thaw cycles and the presence of industrially important enzymes. Extended genomic insights further provided a glimpse on crucial genes likely to be associated with the strategies to adapt harsh environment of high elevation.

## Additional files

Additional file 1: **Figure S1.** Multilocus sequence analysis (MLSA) clustering based phylogenetic tree of six concatenated housekeeping genes as derived from the whole genome sequence from the strains of *Janthinobacterium.* The tree was constructed using the neighbor-joining method based on the JTT matrix-based model using MEGA7 .Bootstrap values over 50% (1000 replications) were shown at each node. All positions containing gaps and missing data were eliminated. The clustering patterns are in agreement with the data generated by the maximum likelihood method. (PDF 238 kb)

Additional file 2: **Table S1.** Whole genome sequence-based in silico comparison of strain ERGS5:01 and other related *Janthinobacterium* strains in database for DDH and ANI. (DOCX 17 kb)

Additional file 3: **Figure S2.** Certificate of deposition of strainERGS5:01 at Microbial Culture Collection (MCC) at National Centre for Cell Science, Pune, India. (PDF 377 kb)

Additional file 4: **Table S2.** Genes predicted to encode industrially important enzymes in the genome of *J. lividum* ERGS5:01. (DOCX 14 kb)

Additional file 5: **Figure S3.** Pan genome analysis of genus *Janthinobacterium*. The pan-genome profile plot displaying the total and the core gene families for each genome with a curve fit exponent of 0.43. [Number of unique genes observed for strains are displayed in brackets; 1. *J. lividum* ERGS5:01 (236); 2. *J.* sp. 551a (0); 3. *J. agaricidamnosum* DSM 9628 (1412); 4.*J.* sp. CG23_2 (2860); 5*. J.* sp. CG3(1038); 6. *J.* sp. HH01 (1764) 7.*J.* sp. KBS0711 (66) 8. *J. lividum* H-24 (217); 9. *J. lividum* NFR18 (86); 10. *J.* sp. Marseille (1625); 11. *J. lividum*MTR (197) 12.*J.* sp. OK676 (171); 13.*J. lividum* PAMC25724 (152); 14. *J. psychotolerans* S3–2 (644); 15.*J.* sp. RA13 (170); 16. *J. lividum* RIT308 (75); 17.*J.* sp. 344 (11); 18.*J.* sp. Ant5–2-1 (136); 19.*J.* sp. HH100 (15); 20.*J.* sp. HH102 (72); 21.*J.* sp._HH103 (16); 22.*J.* sp. HH104 (114); 23.*J.* sp. HH106 (100); 24.*J.* sp. HH107 (78); 25.*J.* sp. MP5059B (136); 26.*J.* sp. TND4EL3 (504); 27.*J.* sp. YR213 (92)]. (PDF 85 kb)

Additional file 6: **Table S3.** List of genes encoding proteins associated with cold adaptation from strain ERGS5:01 among the 1066 core genomes of 27 strain within genus *Janthinobacterium (DOCX 14 kb)*

Additional file 7: **Table S4.** List of genes among the core genomes of 27 *Janthinobacterium* strains associated with general COG functional categories (DOCX 14 kb)

## Abbreviations

ABM: Antarctic Bacterial Medium
ANI: Average nucleotide identities
*aroE*: shikimate dehydrogenase
CSPs: Cold shock proteins
DDH: DNA-DNA hybridization
*gmk*: guanylate kinase
*gyrB*: DNA gyrase B subunit
IMG: Integrated Microbial Genomes
MCC: Microbial Culture Collection
MLSA: Multilocus sequence analysis
MR-VP: Methyl Red and Voges-Proskauer
PacBio: Pacific Biosciences
*RecA*: Recombinase A
*rpoB*: DNA-directed RNA polymerase β subunit
*tpi*: triosephosphate isomerase
