# Supplementary figures and images for: Strategies for high-altitude adaptation revealed from high-quality draft genome of non-violacein producing Janthinobacterium lividum ERGS5:01
Source: Stand Genomic Sci. 2018 Apr 19;13:11. doi: 10.1186/s40793-018-0313-3 (PMC5909252; doi:10.1186/s40793-018-0313-3)

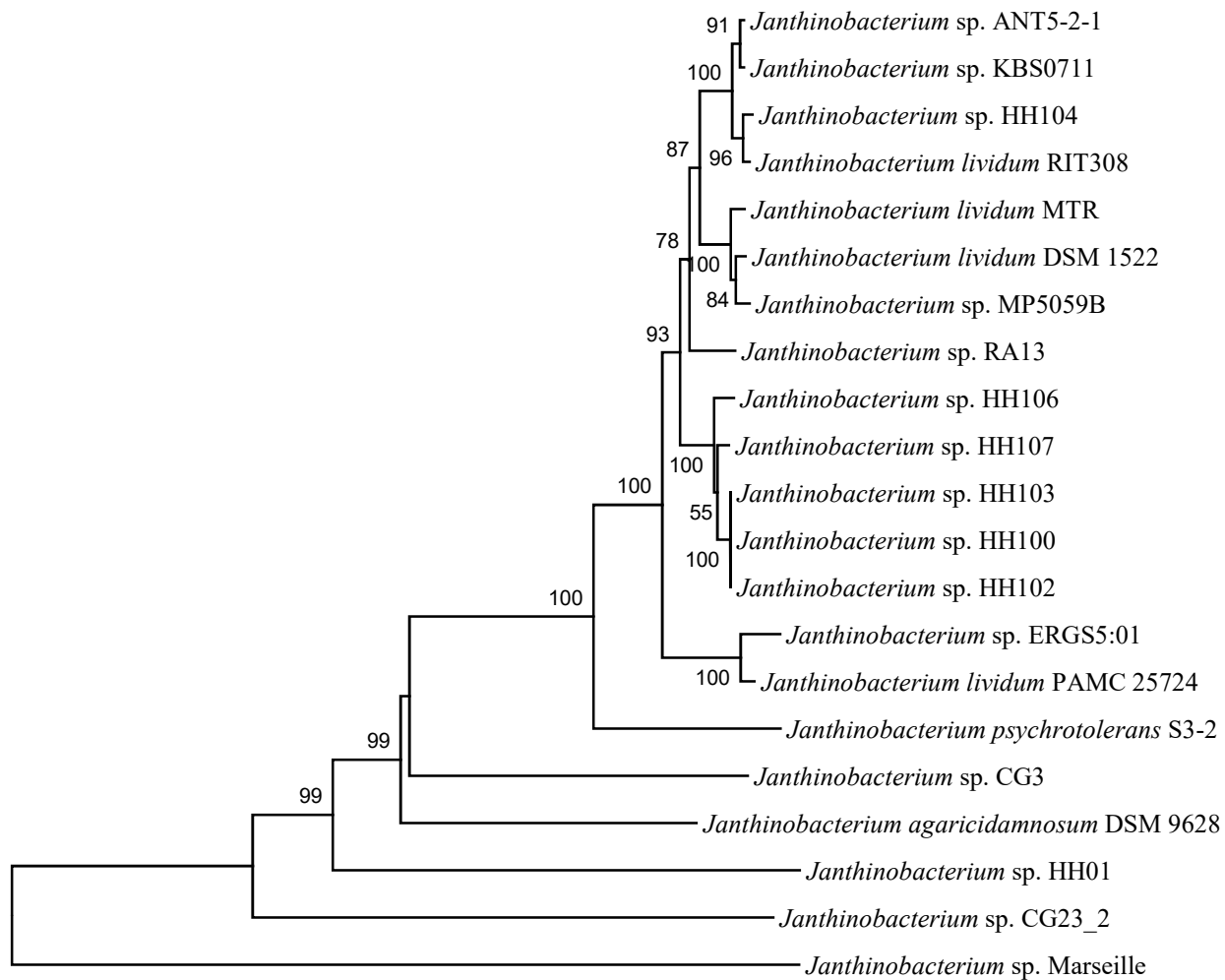

0.020

Supplement: Supplementary file 1 — Figure S1. Multilocus sequence analysis (MLSA) clustering based phylogenetic tree of six concatenated housekeeping genes as derived from the whole genome sequence from the strains of Janthinobacterium. The tree was constructed using the neighbor-joining method based on the JTT matrix-based model using MEGA7 .Bootstrap values over 50% (1000 replications) were shown at each node. All positions containing gaps and missing data were eliminated. The clustering patterns are in agreement with the data generated by the maximum likelihood method. (PDF 238 kb) [file 40793_2018_313_MOESM1_ESM.pdf]
