# Supplementary material for: Strategies for high-altitude adaptation revealed from high-quality draft genome of non-violacein producing Janthinobacterium lividum ERGS5:01
Source: Stand Genomic Sci. 2018 Apr 19;13:11. doi: 10.1186/s40793-018-0313-3 (PMC5909252; doi:10.1186/s40793-018-0313-3)
Supplement: Supplementary file 2 — Table S1. Whole genome sequence-based in silico comparison of strain ERGS5:01 and other related Janthinobacterium strains in database for DDH and ANI. (DOCX 17 kb) [file 40793_2018_313_MOESM2_ESM.docx]

**Table S1.** Whole genome sequences based *in silico* comparison of strain ERGS5:01 and other related *Janthinobacterium* strains in database for DDH and ANI.

| Sl. No. | Strain | ANI (%) | DDH (%) (Model-based  Confidence Interval) (%) |
| --- | --- | --- | --- |
| 1 | *Janthinobacterium lividum* PAMC 25724 | 99.25 | 95.10 (93.5 - 96.3) |
| 2 | *Janthinobacterium lividum* H-24 (DSM 1522) | 89.53 | 38.30 (35.9 - 40.9) |
| 3 | *Janthinobacterium lividum* MTR | 89.53 | 38.70 (36.3 - 41.3) |
| 4 | *Janthinobacterium lividum* RIT308 | 89.15 | 37.70 (35.3 - 40.2) |
| 5 | *Janthinobacterium* sp. Ant5-2-1 | 89.00 | 38.80 (36.4 - 41.4) |
| 6 | *Janthinobacterium* sp. 344 | 87.56 | 34.20 (31.8 - 36.7) |
| 7 | *Janthinobacterium* sp. HH100 | 89.56 | 38.00 (35.6 - 40.6) |
| 8 | *Janthinobacterium* sp. HH102 | 89.511 | 38.00 (35.6 - 40.5) |
| 9 | *Janthinobacterium* sp. HH103 | 89.30 | 38.00 (35.6 - 40.6) |
| 10 | *Janthinobacterium* sp. HH104 | 89.01 | 37.50 (35.1 - 40) |
| 11 | *Janthinobacterium* sp. HH106 | 89.32 | 38.10 (35.6 - 40.6) |
| 12 | *Janthinobacterium* sp. HH107 | 89.35 | 38.10 (35.6 - 40.6) |
| 13 | *Janthinobacterium lividum* NFR18 | 89.13 | 37.60 (35.1 - 40.1) |
| 14 | *Janthinobacterium* sp. OK676 | 88.85 | 37.20 (34.8 - 39.7) |
| 15 | *Janthinobacterium* sp. 551a | 87.67 | 34.20 (31.8 - 36.7) |
| 16 | *Janthinobacterium* sp. RA13 | 89.28 | 38.10 (35.6 - 40.6) |
| 17 | *Janthinobacterium* sp. KBS0711 | 88.64 | 37.80 (35.3 - 40.3) |
| 18 | *Janthinobacterium* sp. MP5059B | 89.47 | 38.60 (36.2 - 41.2) |
| 19 | *Janthinobacterium* sp. TND4EL3 | 88.33 | 35.90 (33.5 - 38.4) |
| 20 | *Janthinobacterium* sp. YR213 | 89.49 | 38.50 (36 - 41) |
| 21 | *Janthinobacterium psychotolerans* S3-2 | 83.15 | 26.80 (24.4 - 29.3) |
| 22 | *Janthinobacterium* sp. Marseille | 72.51 | 21.00 (18.8 - 23.4) |
| 23 | *Janthinobacterium agaricidamnosum*  DSM 9628 | 78.86 | 23.00(20.7 - 25.5) |
| 24 | *Janthinobacterium* sp. CG23 | 75.60 | 21.20(19 - 23.7) |
| 25 | *Janthinobacterium* sp. CG3 | 78.58 | 22.60(20.4 - 25.1) |
| 26 | *Janthinobacterium* sp. HH01 | 74.77 | 21.80 (19.6 - 24.3) |
