# Supplementary material for: Strategies for high-altitude adaptation revealed from high-quality draft genome of non-violacein producing Janthinobacterium lividum ERGS5:01
Source: Stand Genomic Sci. 2018 Apr 19;13:11. doi: 10.1186/s40793-018-0313-3 (PMC5909252; doi:10.1186/s40793-018-0313-3)
Supplement: Supplementary file 3 — Figure S2. Certificate of deposition of strainERGS5:01 at Microbial Culture Collection (MCC) at National Centre for Cell Science, Pune, India. (PDF 377 kb) [file 40793_2018_313_MOESM3_ESM.pdf]

MCC

सुक्ष्मजीव संवर्धन संकलन
MICROBIAL CULTURE COLLECTION
Member of WFCC, An IDA under Budapest Treaty, DNR of MoEF
राष्ट्रीय कोशिका विज्ञान केंद्र | National Centre for Cell Science

Certificate Of Deposit

Janthinobacterium lividum **strain ERGS5:01**

was received for public access deposit at MCC from

Dr. Rakshak Kumar

Biotechnology Division, CSIR Institute of Himalayan Bioresource Technology

Palampur 176 061, Himachal Pradesh, India

on **26 October 2015**

and was allocated the accession number

MCC 2953

After confirming the viability, purity and authenticity of the strain in the MCC facilities, it has been preserved using standard methods.

The strain is now available to the public.

Dr. Amit Yadav

Scientist, Microbial Culture Collection, Pune

19 January 2016

साई ट्रिनिटी कॉम्प्लेक्स, सुस रोड, पाषाण, पुणे-४११ ०२१. महाराष्ट्र, भारत.
Sai Trinity Complex, Sus Road, Pashan, Pune- 411 021. Maharashtra, INDIA
दुरभाष / Tel: +9120 25329000| फ़ैक्स / Fax: +9120 25329001
ई-मेल / e-mail: mcc@nccs.res.in | वेब / Web: www.nccs.res.in/mcc
एनसीसीएस भारत सरकार के जैवप्रौद्योगिकी विभाग का स्वायत्त संस्थान है.
NCCS is an Autonomous Institute of Department of Biotechnology, Govt. of India
