# Supplementary material for: Strategies for high-altitude adaptation revealed from high-quality draft genome of non-violacein producing Janthinobacterium lividum ERGS5:01
Source: Stand Genomic Sci. 2018 Apr 19;13:11. doi: 10.1186/s40793-018-0313-3 (PMC5909252; doi:10.1186/s40793-018-0313-3)
Supplement: Supplementary file 4 — Table S2. Genes predicted to encode industrially important enzymes in the genome of J. lividum ERGS5:01. (DOCX 14 kb) [file 40793_2018_313_MOESM4_ESM.docx]

| **Industrially important enzymes** | **GenBank ID** |
| --- | --- |
| Alpha-amylase | OFJ46974 |
| Lipase | OFJ49912, OFJ46835, OFJ46836 |
| Serine protease | OFJ47175 |
| Phospholipase | OFJ47411, OFJ48321, OFJ48322 |
| Glycosyl transferase | OFJ50221,OFJ50223, OFJ49554, OFJ50261,OFJ49903, OFJ46384, OFJ46432, OFJ46439, OFJ49303 |
| Alcohol dehydrogenase | OFJ47191, OFJ48538, OFJ48571, OFJ48888 |
| Catalase | OFJ46378 |
| Alkaline phosphatase | OFJ50097, OFJ47066, OFJ48452, OFJ49083 |
| Chitinase | OFJ50269, OFJ47640 |

**Table S2.** List of predicted genes encoding industrially important enzymes in the genome of [J. lividum](http://doi.org/10.1601/nm.1711) ERGS5:01.
