# Supplementary material for: Strategies for high-altitude adaptation revealed from high-quality draft genome of non-violacein producing Janthinobacterium lividum ERGS5:01
Source: Stand Genomic Sci. 2018 Apr 19;13:11. doi: 10.1186/s40793-018-0313-3 (PMC5909252; doi:10.1186/s40793-018-0313-3)
Supplement: Supplementary file 5 — Figure S3. Pan genome analysis of genus Janthinobacterium. The pan-genome profile plot displaying the total and the core gene families for each genome with a curve fit exponent of 0.43. [Number of unique genes observed for strains are displayed in brackets; 1. J. lividum ERGS5:01 (236); 2. J. sp. 551a (0); 3. J. agaricidamnosum DSM 9628 (1412); 4.J. sp. CG23_2 (2860); 5. J. sp. CG3(1038); 6. J. sp. HH01 (1764) 7.J. sp. KBS0711 (66) 8. J. lividum H-24 (217); 9. J. lividum NFR18 (86); 10. J. sp. Marseille (1625); 11. J. lividumMTR (197) 12.J. sp. OK676 (171); 13.J. lividum PAMC25724 (152); 14. J. psychotolerans S3–2 (644); 15.J. sp. RA13 (170); 16. J. lividum RIT308 (75); 17.J. sp. 344 (11); 18.J. sp. Ant5–2-1 (136); 19.J. sp. HH100 (15); 20.J. sp. HH102 (72); 21.J. sp._HH103 (16); 22.J. sp. HH104 (114); 23.J. sp. HH106 (100); 24.J. sp. HH107 (78); 25.J. sp. MP5059B (136); 26.J. sp. TND4EL3 (504); 27.J. sp. YR213 (92)]. (PDF 85 kb) [file 40793_2018_313_MOESM5_ESM.pdf]

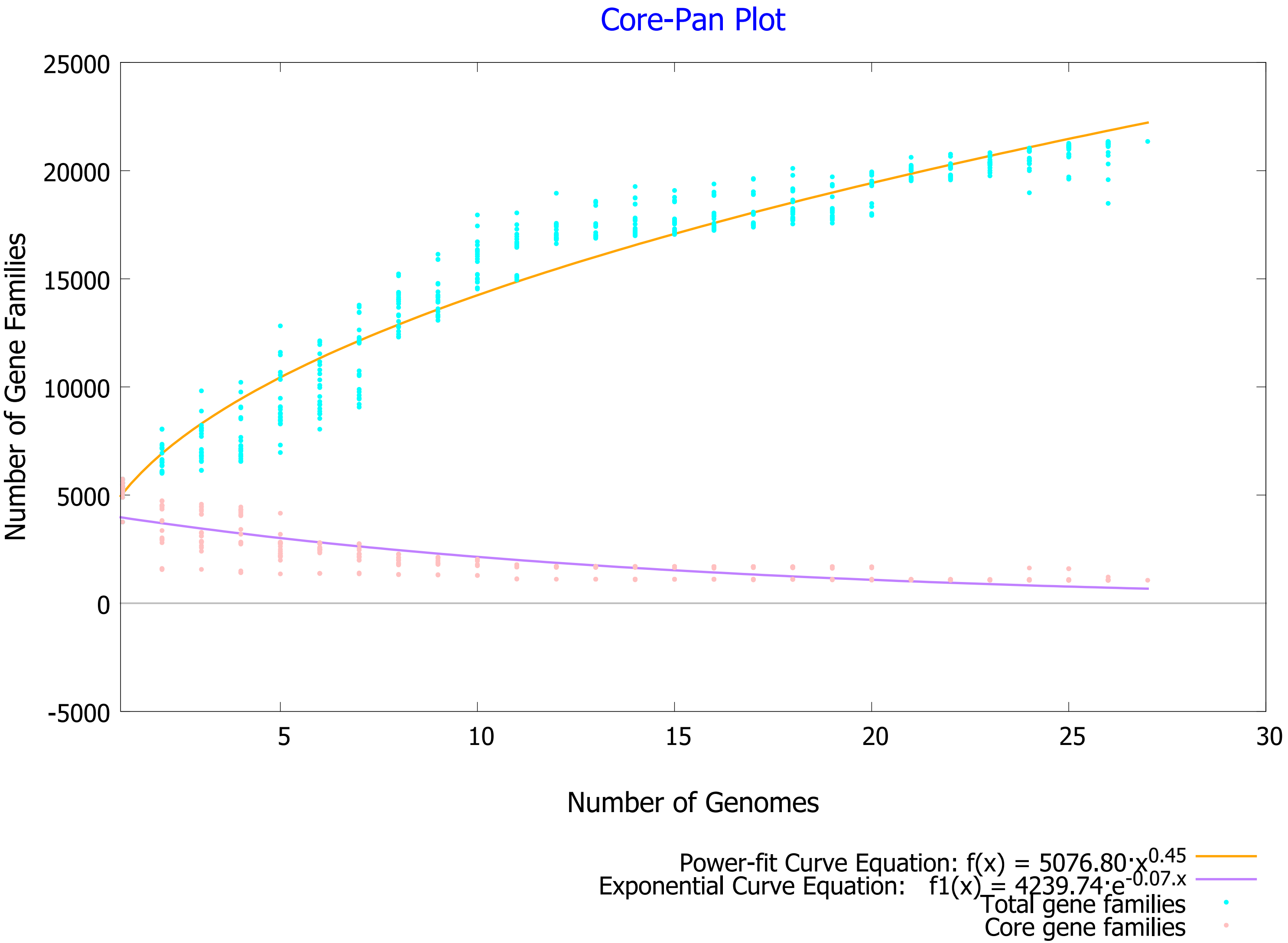
Core-Pan Plot
Number of Gene Families
25000
20000
15000
10000
5000
0
-5000
5
10
15
20
25
30
Number of Genomes
Power-fit Curve Equation: f(x) = 5076.80·x^0.45
Exponential Curve Equation: f1(x) = 4239.74·e^-0.07.x
Total gene families
Core gene families
