# Supplementary material for: Strategies for high-altitude adaptation revealed from high-quality draft genome of non-violacein producing Janthinobacterium lividum ERGS5:01
Source: Stand Genomic Sci. 2018 Apr 19;13:11. doi: 10.1186/s40793-018-0313-3 (PMC5909252; doi:10.1186/s40793-018-0313-3)
Supplement: Supplementary file 6 — Table S3. List of genes encoding proteins associated with cold adaptation from strain ERGS5:01 among the 1066 core genomes of 27 strain within genus Janthinobacterium (DOCX 14 kb) [file 40793_2018_313_MOESM6_ESM.docx]

| **Category and Gene ID** | **Description** |
| --- | --- |
| **Two component sensor histidine kinase** | |
| OFJ50245, OFJ47405 | two-component sensor histidine kinase |
| OFJ49383 | two-component system sensor histidine kinase KdbD |
| OFJ46773, OFJ49454 | histidine kinase |
| **Cold-shock** | |
| OFJ46770 | cold-shock protein |
| **DNA repair** | |
| OFJ46839 | transcription-repair coupling factor |
| OFJ47392 | DNA repair protein RadA |
| OFJ47351 | DNA repair protein RecO |
| OFJ49466. | DNA mismatch repair protein MutL |
| OFJ47699 | DNA mismatch repair protein MutS |
| **Cold-active chaperones** | |
| OFJ47043 | ATP-dependent chaperone ClpB |
| OFJ47490 | Fe-S protein assembly chaperone HscA |
| OFJ49231 | molecular chaperone DnaK |
| OFJ47087 | molecular chaperone HtpG |
| OFJ49232 | molecular chaperone DnaJ |
| OFJ47491 | Fe-S protein assembly co-chaperone HscB |
| OFJ49967 | RNA chaperone Hfq |
| OFJ48758 | protein-export chaperone SecB |
| OFJ49648. | co-chaperone GroES |
| OFJ49647 | chaperonin GroL |
| OFJ47087 | molecular chaperone HtpG |
| **Oxidative stress** | |
| OFJ47698 | thioredoxin-disulfide reductase |
| OFJ47367 | thiol reductase thioredoxin |
| OFJ49306. | thioredoxin |
| OFJ49418 | alkyl hydroperoxide reductase |
| OFJ49024 | alkyl hydroperoxide reductase subunit F |
| OFJ48937 | organic hydroperoxide resistance protein |
| OFJ48027 | superoxide dismutase |
| **Carbon storage/starvation** | |
| OFJ47264 | glycerol acyltransferase |
| OFJ49489 | Glycerol-3-phosphate acyltransferase |
| **Membrane/ cell wall alteration** | |
| OFJ47939 | UDP-N-acetylglucosamine 1-carboxyvinyltransferase |
| OFJ47361 | 3-oxoacyl-[acyl-carrier-protein] synthase |
| OFJ47939 | UDP-N-acetylenolpyruvoylglucosamine reductase |

**Table S3.** List of genes encoding proteins associated with cold adaptation from strain ERGS5:01 among the 1066 core genomes of 27 strain within genus *Janthinobacterium*
