# Supplementary material for: Strategies for high-altitude adaptation revealed from high-quality draft genome of non-violacein producing Janthinobacterium lividum ERGS5:01
Source: Stand Genomic Sci. 2018 Apr 19;13:11. doi: 10.1186/s40793-018-0313-3 (PMC5909252; doi:10.1186/s40793-018-0313-3)
Supplement: Supplementary file 7 — Table S4. List of genes among the core genomes of 27 Janthinobacterium strains associated with general COG functional categories (DOCX 14 kb) [file 40793_2018_313_MOESM7_ESM.docx]

| **Code** | **Value** | **%age** | **Description** |
| --- | --- | --- | --- |
| J | 152 | 14.3% | Translation, ribosomal structure and biogenesis |
| A | 1 | 0.09% | RNA processing and modification |
| K | 152 | 14.3% | Transcription |
| L | 68 | 6.4% | Replication, recombination and repair |
| B | 0 | 0.00% | Chromatin structure and dynamics |
| D | 22 | 2.1% | Cell cycle control, Cell division, chromosome partitioning |
| V | 35 | 3.3% | Defense mechanisms |
| T | 187 | 17.5% | Signal transduction mechanisms |
| M | 125 | 11.7% | Cell wall/membrane biogenesis |
| N | 92 | 8.6% | Cell motility |
| U | 37 | 3.5% | Intracellular trafficking and secretion |
| O | 97 | 9.1% | Posttranslational modification, protein turnover, chaperones |
| C | 118 | 11.1% | Energy production and conversion |
| G | 81 | 7.6% | Carbohydrate transport and metabolism |
| E | 167 | 15.7% | Amino acid transport and metabolism |
| F | 54 | 5.1% | Nucleotide transport and metabolism |
| H | 118 | 11.1% | Coenzyme transport and metabolism |
| I | 105 | 9.8% | Lipid transport and metabolism |
| P | 95 | 8.9% | Inorganic ion transport and metabolism |
| Q | 34 | 3.2% | Secondary metabolites biosynthesis, transport and catabolism |
| R | 139 | 13.0% | General function prediction only |
| S | 56 | 5.33% | Function unknown |
| - | 53 | 5.0% | Not in COGs |

**Table S 4**. List of genes among the core genomes of 27 *Janthinobacterium* strains associated with general COG functional categories
